# The lipid lowering efficacy of PCSK9 inhibitors alone vs. statins alone: a meta-analysis

**DOI:** 10.3389/fcvm.2026.1769430

**Published:** 2026-02-17

**Authors:** Katie Kyan, Jeffrey Gornbein, Jeffrey Saver

**Affiliations:** 1 California University of Science and Medicine, Colton, CA, United States; 2 Statistics Core, Department of Medicine, David Geffen School of Medicine, UCLA, Los Angeles, CA, United States; 3 Comprehensive Stroke Center and Department of Neurology, David Geffen School of Medicine, UCLA, Los Angeles, CA, United States

**Keywords:** atherosclerosis, hyperlipidemia, LDL cholesterol, lipid lowering agent, PCSK9 inhibitors, statins

## Abstract

**Background:**

PCSK9 inhibitors (PCSK9is) have been developed as an add-on therapy to maximally tolerated statin therapy. To date, high prices have precluded their use as first-line agents but, in the near future, PCSK9is will go off patent protection, reducing cost barriers and making them first-line agent candidates before statins. This study's objective was to evaluate the lipid lowering efficacy of PCSK9i monotherapy compared with high intensity statin monotherapy as a first line agent.

**Methods:**

This meta-analysis adhered to PRISMA guidelines. A systematic literature review identified all RCTs of: 1) PCSK9i vs. control in which data were available on a patient subgroup receiving PCSK9i monotherapy (predominantly due to total statin intolerance); and 2) high-intensity statins vs. control, with high-intensity statins defined as atorvastatin 40 or 80 mg daily or rosuvastatin 20 or 40 mg daily. The primary outcome was mean percent change in serum low density lipoprotein cholesterol (LDL-C). Secondary outcomes evaluated high density lipoprotein cholesterol (HDL-C), total cholesterol (TC), triglycerides (TG), and apolipoprotein B (ApoB).

**Results:**

Five trials (766 patients) were identified for PCSK9is and 49 trials (19,603 patients) for statins. The mean age (±SD) was 57.5 ± 5.1 years in PCSK9i trials and 60.2 ± 2.3 years in statin trials, with a higher proportion of women enrolled in the PCSK9i group (54.3% vs. 39.9%). Compared to all high-intensity statin regimens combined, PCSK9is showed significantly greater reductions in LDL-C (−52.4% vs. −46.6%, *p* = 0.03), ApoB (−43.3% vs. −32.8%, *p* = 0.004), and increases in HDL-C (8.0% vs. 4.4%, *p* = 0.02). Statin agent subgroup analysis found PCSK9is were superior to atorvastatin and comparable to rosuvastatin in increasing HDL-C and reducing LDL-C, ApoB, and TC, and inferior to atorvastatin and rosuvastatin in reducing TG.

**Conclusions:**

PCSK9i monotherapy is superior to atorvastatin and comparable to rosuvastatin in improving LDL-C, HDL-C, TC, and ApoB, though inferior in reducing TG. These findings confirm that PCSK9is are currently highly useful second-line agents in patients with total statin intolerance and in the near future, after expiration of patent protection, will be useful first line agents for hyperlipidemia.

## 1 Introduction

Cardiovascular disease (CVD) has remained the leading cause of death in the United States since 1921 (1). Despite a decline in CVD over the past few decades, recent data from The American Heart Association shows that death rates due to CVD have started to increase yet again (1). They have also reported that stroke is the fifth leading cause of death in the United States and the number of deaths due to stroke increased by 26.3% from 2011 to 2021. Both CVD and stroke are among the leading causes of death worldwide and are tied to many of the same risk factors such as hyperlipidemia. Studies have shown that hyperlipidemia is considered an independent predictor of cardiovascular and cerebrovascular events including myocardial infarction and stroke (2). This is because increased levels of low-density lipoprotein (LDL) are particularly harmful as LDL is rich in cholesterol and a buildup can contribute to the formation of atherosclerotic plaques.

Statins, which are competitive inhibitors of hydroxymethylglutaryl-CoA reductase, play a key role in reducing low-density lipoprotein cholesterol (LDL-C) levels. Randomized clinical trials have demonstrated that statins reduce the mortality and morbidity rates of patients with stroke and CVD (2). As such, statins have long been considered the first line treatment for hyperlipidemia and have been commonly used since the mid-1980s (3). However, statins are not tolerated by all patients and can lead to side effects such as myalgia.

Proprotein convertase subtilisin/kexin type 9 inhibitors (PCSK9is) are another group of lipid-lowering drugs that decrease LDL-C levels and have been shown to reduce the risk of stroke and CVD. They are frequently used as an add-on therapy for patients with suboptimal LDL-C levels who are also taking maximally tolerated statins (3). Although PCSK9is can be taken alone, their high price precludes their use as first-line agents. In fact, when PCSK9is first arrived to the market in 2015, the cost was approximately $15,000 per year (4). In 2019, the cost of PCSK9is was reduced to approximately $5850 per year, but these prices still remain a significant barrier for many patients.

However, in the near future, PCSK9is will go off patent protection and their price will be reduced. At that time, PCSK9is may be used as first-line agents to treat hyperlipidemia before statins. Therefore, it is of interest to compare the efficacy of PCSK9is as a first line agent directly with statins as a first line agent. We conducted a meta-analysis to evaluate the lipid-lowering efficacy of these drugs and to better understand their effects on reducing cardiovascular and stroke outcomes. In doing so, we also aim to characterize the effects of PCSK9is as a first line treatment to provide clinicians with information about the effects they should expect in patients who are statin intolerant.

## 2 Methods

We performed a systematic review and meta-analysis of PCSK9 inhibitor and statin randomized controlled trials (RCTs) in order to evaluate the lipid lowering efficacy of each drug class. This review was conducted in accordance with the Preferred Reporting Items of Systematic Reviews and Meta-Analyses (PRISMA) statement (5).

### 2.1 Meta-analysis of PCSK9i vs. control trials

#### 2.1.1 Identifying PCSK9i vs. control trials

To gather data from randomized trials comparing PCSK9is without statins to controls without statins, we first systematically searched PubMed for recent meta-analyses on PCSK9is from January 2023 to December 2024 using the keywords (“pcsk9” or “pcsk-9” or “proprotein convertase subtilisin/kexin type 9”) and “cardiovascular” and “systematic review” and “meta-analysis”. RCTs assessing PCSK9is were identified by extraction from the retrieved meta-analyses. We also searched for any RCTs published after the final search date of the most recent meta-analysis, from July 2024 to December 2024, using the keywords (“pcsk9” or “pcsk-9” or “proprotein convertase subtilisin/kexin type 9”) and “cardiovascular” and “randomized” or “trial”.

#### 2.1.2 Study selection

Criteria for inclusion of a study were: (1) RCT or meta-analysis of RCTs of PCSK9i; and (2) LDL-C lowering magnitude data were available from the full trial population or an identifiable subset of the trial population on patients taking PCSK9is without any concomitant statin therapy. Exclusion criteria were: (1) Reported outcome data did not include magnitude of serum lipid-lowering; (2) data for lipid-lowering efficacy was reported only for patients taking PCSK9is but not controls; (3) patient population was receiving lipid-lowering therapy for a condition other than the primary or secondary prevention of cardiovascular disease; (4) studied agent targeted long-term lipid lowering (siRNA PCSK9i) rather than short- and long-term lipid lowering (evolucumab and alirocumab); (5) studied agent was a PCSK9i not FDA-approved; (6) article not in English; and (7) small sample size (under 50). One reviewer screened all studies (K.A.K.), and a second reviewer (J.L.S.) screened the studies that were deemed as qualifying by the first reviewer. Any disagreements were addressed by consensus discussion.

### 2.2 Meta-analysis of high intensity statin trials

#### 2.2.1 Identifying high intensity statin vs. control trials

Statin regimens considered high-intensity were rosuvastatin 20 mg–40 mg daily or atorvastatin 40 mg–80 mg daily (6). We systematically searched PubMed for meta-analyses on high-intensity statins using the keywords “statin” and (“rosuvastatin” or “atorvastatin” or “intensive” or “high-intensity” or “high intensity”) and “efficacy” and (“comparison” or “comparative”) and “meta-analysis”. RCTs assessing PCSK9is were identified by extraction from the retrieved meta-analyses. We also searched for any RCTs published after the final search date of the most recent meta-analysis, from July 2024 to December 2024, using the keywords “statin” and (“rosuvastatin” or “atorvastatin” or “intensive” or “high-intensity” or “high intensity”) and “efficacy” and (“comparison” or “comparative”) and (“randomized” or “trial”).

#### 2.2.2 Study selection

Criteria for inclusion of a study were: (1) RCT of high intensity statins (rosuvastatin 20–40 mg and atorvastatin 40–80 mg); and (2) LDL-C lowering magnitude data were available from the full trial population or an identifiable subset of the trial population on patients taking high intensity statins without any concomitant PCSK9i therapy. Exclusion criteria were: (1) reported outcome data did not include magnitude of serum lipid-lowering; (2) data for lipid-lowering efficacy was reported only for patients taking statins but not controls; (3) patient population was receiving lipid-lowering therapy for a condition other than the primary or secondary prevention of cardiovascular disease; (4) studied agent was a statin not FDA-approved; (5) lipid-lowering results were missing information required for mathematical poolability (mean value with SD, SE, and/or Cis) (6) small sample size (under 50); (7) study population had only short-term, procedural indication for statins (e.g., CABG, PCI, cardiac bypass) (8); papers evaluating evaluating combinations of statins rather than only one high intensity statin (9) a substantial proportion (≥25%) of the study population was outside of North America and/or Europe; (11) treat to target trials in which some patients received doses of statins below the high intensity range; and (12) article not in English. The exclusion of studies with substantial members of non-North America, non-European populations was made to enhance comparability of study populations as the PCSK9i trials were conducted primarily in North America and Europe. One reviewer screened all studies (K.A.K.), and a second reviewer (J.L.S.) screened the studies that were deemed as qualifying by the first reviewer. Any disagreements were addressed by consensus discussion.

### 2.3 Data extraction

All qualifying full-text manuscripts were reviewed and data extracted by 2 independent investigators (K.A.K. and J.L.S.), and discrepancies were resolved by discussion.

From all qualifying trials, we extracted the following baseline data values about the patient populations: Number of participants, age, sex, race (white, black, asian, other race, non-white, and hispanic), hypertension, diabetes mellitus, tobacco use, body mass index (BMI), LDL-C, high density lipoprotein cholesterol (HDL-C), total cholesterol (TC), triglycerides (TG), and apolipoprotein B (ApoB).

To evaluate the efficacy measures from these trials, we extracted data on the mean percentage change for the following: LDL-C, TC, TG, HDL-C, and ApoB. For all trials, results were extracted only from the active treatment arms that met our pre-specified definitions for high-intensity statin or PCSK9i monotherapy. Data was not extracted from control arms. In trials testing multiple statin doses, only the arms meeting the high-intensity threshold were included.

### 2.4 Risk of bias

For study-level data meta-analyses, the risk of bias (e.g., randomization process, intended interventions, missing outcome data, measurement of the outcome, and selection of the reported result) for each trial was assessed using the revised Cochrane tool for assessing risk of bias in randomised trials also known as RoB 2 (7). The overall risk of bias was then classified as high risk, some concern, and low risk using the established criteria. For individual participant-level data meta-analyses, risk of bias was not formally assessed due to the absence of appropriate rating tools (8).

### 2.5 Statistical method

The percent change standard deviation (SD) or standard error (SE) was not reported in some of the 18 studies. The ideal meta-analysis weights are the inverse of the squared SE of the mean percent change which is computed from the SD divided by the square root of *n* [SE = SD/sqrt(n)]. This percent change SD is in turn approximately a function of the pre mean, pre-SD, post mean, post-SD and the correlation (r) between pre vs. post outcome values. In order to compute the SD percent change and corresponding SE, the below formula was solved for r and the values of r were computed for the groups where the percent change SD was reported.

$$\mathrm{percent}\;\mathrm{change}\;\mathrm{SD}\approx 100\times \surd [M^{2}\times ({CV}_{1}^{2}+{CV}_{2}^{2}-2r(CV_{1}\times CV_{2}))]$$

where M = post-mean/pre-mean, CV_1_ = pre-SD/pre-mean, CV_2_ = post-SD/post-mean.

The median correlation value for a given outcome was used to compute the missing percent change SD where needed. The actual summary mean and its standard error across studies was computed using the DerSimonian-Laird (DL) random effects model (R package “metafor”-DerSimonian & Laird, 1986; Raudenbush, 2009). Dr. Saver had full access to all the data in the study and takes responsibility for its integrity and the data analysis.

## 3 Results

### 3.1 Selection of PCSK9i vs. control trials

**Figure 1:**
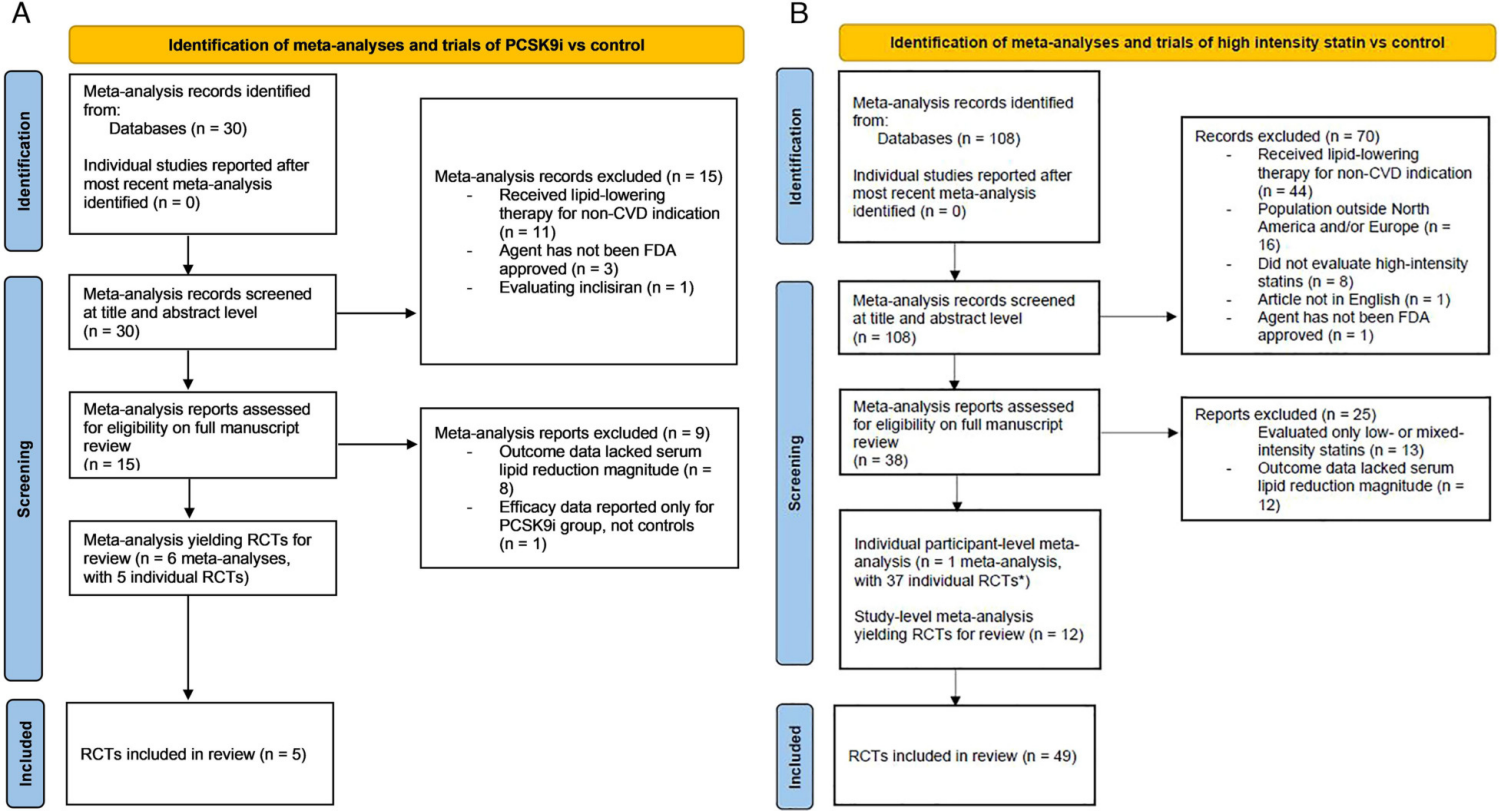
PRISMA flow diagrams. **(A)** Identification of Meta-Analyses and Trials of High Intensity Statin vs. Control. Article selection flow for meta-analysis of trials of high-intensity statins vs control reporting effects on lipid levels. PRISMA indicates Preferred Reporting Items for Systematic Reviews and Meta-Analyses; and RCT, randomized controlled trial. **(B)** Identification of Meta-Analyses and Trials of PCSK9i vs. Control. Article selection flow for meta-analysis of trials of PCSK9 inhibitors vs control reporting effects on lipid levels. *Study records identified from most recent comprehensive meta-analysis (VOYAGER 2010). PRISMA indicates Preferred Reporting Items for Systematic Reviews and Meta-Analyses; and RCT, randomized controlled trials.

Systematic search yielded a total of 30 meta-analyses, among which 15 records were excluded after screening study titles or abstracts and 9 further records excluded upon detailed reading of the full text. The leading reasons for exclusion were: patient population was receiving lipid-lowering therapy for a condition other than the primary or secondary prevention of cardiovascular disease (*n* = 11); and reported outcome data did not include magnitude of serum lipid-lowering (*n* = 8). From the 6 meta-analyses meeting inclusion criteria, we identified 5 RCTS in which PCSK9i outcomes were evaluated in a subgroup of patients who were not on any statin treatment (9–13). No later trials reported after those already included in one of the meta-analyses were identified (Figure 1A). The risk of bias was assessed as low for three studies and high for two studies (Table 1).

**Table 1:** Risk of bias assessments.

Study Name	D1^a^	D2^a^	D3^a^	D4^a^	D5^a^	Overall
High Intensity Statin Trials^b^						
SPARCL 2006 (14)	S	L	L	L	L	S
Betteridge et al. 2007 (15)	L	L	S	L	L	S
Capuzzi et al. 2003 (16)	L	S	H	L	L	H
Catapano et al. 2006 (17)	L	L	H	L	L	H
Conard et al. 2008 (18)	L	L	S	L	L	S
DALI 2001 (19)	L	L	H	L	L	H
Lawrence et al. 2004 (20)	L	L	H	L	L	H
Leiter et al. 2008 (21)	L	L	S	L	L	S
SATURN 2011 (22)	L	L	H	L	L	H
Olsson et al. 2001 (24)	L	H	H	L	L	H
IDEAL 2005 (25)	L	L	L	L	L	L
Schneider et al. 2004 (26)	L	L	L	L	L	L
**PCSK9i Trials**						
MENDEL-2 2014 (9)	L	L	L	L	L	L
MENDEL 2012 (10)	L	L	L	L	L	L
GAUSS-3 2016 (11)	L	L	H	L	L	H
ODYSSEY MONO 2014 (12)	L	L	H	L	L	H
ODYSSEY CHOICE 2016 (13)	L	L	L	L	L	L

L, indicates low risk; S, some concerns; H, high risk.

^a^ D1 = Risk of bias arising from the randomization process; D2 = Risk of bias due to deviations from the intended interventions; D3 = Risk of bias due to missing outcome data; D4 = Risk of bias in measurement of the outcome; D5 = Risk of bias in selection of the reported result.

^b^ Risk of bias for the data from the VOYAGER 2010 meta-analysis not formally assessed due to absence of appropriate bias rating tools for individual participant-level data meta-analyses.

### 3.2 Selection of high intensity statin vs. control trials

Systematic search yielded a total of 108 meta-analyses, among which 70 were excluded after screening study titles or abstracts, and 38 excluded upon detailed reading of the full text. The leading reasons for exclusion were: patient population was receiving lipid-lowering therapy for a condition other than the primary or secondary prevention of cardiovascular disease (*n* = 44); study population was outside of the North America and/or Europe (*n* = 16); and papers evaluating only low-intensity or mixed-intensity statins rather only one high-intensity statin (*n* = 13). Of the 14 meta-analyses meeting selection criteria, one was an individual participant-level meta-analysis and 13 study-level meta-analyses. Given the better capability for patient characteristic adjustment, the individual participant-level meta-analysis known as VOYAGER (indiVidual patient meta-analysis Of statin therapy in At risk Groups: Effects of Rosuvastatin, atorvastatin and simvastatin) was selected for inclusion, providing 37 trials (23). In the systematic search for more recent individual RCTs, 12 additional trials meeting entry criteria were identified, yielding a total of 49 trials [(14–26); Figure 1B].

### 3.3 Patient characteristics

The final analysis included 5 RCTs of PCSK9is vs inactive control (placebo or open label), comprising a total sample size of 766 patients. For statins, 49 RCTs of high intensity statins vs control (placebo or open label) were included, comprising a total sample size of 19,603 patients. In the statin analysis, 20 data points were analyzed, comprised of 4 active arms from an an individual participant-data level pooled analysis of 37 trials plus 16 active arms from 12 trials not included in the pooled analysis. Baseline characteristics of the enrolled populations are shown in Table 2 (study-level means) and Supplementary Table S1 (sample-size weighted means). Considering study-level means, high intensity statin and PCSK9i trial patients were similar in age, and high intensity statin patients were less often female, less often white, and more often had vascular risk factors of hypertension, diabetes, and tobacco use, but the groups had similar BMIs.

**Table 2:** Baseline characteristics and lipid parameters (study-level means).

Characteristic	PCSK9i Trials	High Intensity Statin Trials
	(5 trials; 766 pts) [n]^a^	(49 trials^c^; 19,603 patients) [n]^a^
Age, mean	57.5 ± 5.1 [5]	60.2 ± 2.3 [48]
Female (%)	54.3 ± 12.5 [5]	39.9 ± 11.0 [48]
BMI (kg/m²)	29.5 ± 1.6 [4]	29.3 ± 1.4 [7]
Race-Ethnicity^b^		
White (%)	88.4 ± 6.1 [5]	73.0 ± 29.9 [43]
Black (%)	7.9 ± 6.9 [3]	7.9 ± 2.1 [40]
Asian (%)	7.8 [1]	6.8 ± 1.7 [38]
Other Race (%)	2.7 ± 2.4 [2]	6.4 ± 5.2 [38]
Hispanic (%)	6.4± N/A [1]	4.3 [1]
Vascular Risk Factors		
Hypertension (%)	43.5 ± 14.4 [3]	61.5 ± 27.0 [6]
Diabetes Mellitus (%)	5.6 ± 7.8 [4]	59.1 ± 38.2 [47]
Tobacco Use (%)	13.6 ± 4.4 [2]	23.5 ± 1.4 [5]
Lipid Parameters		
LDL-C mean (mg/dL)	160.3 ± 33.7 [5]	147.6 ± 34.6 [49]
TC mean (mg/dL)	247.9 ± 40.8 [4]	220.6 ± 30.3 [12]
TG mean (mg/dL)	59.9 [1]	154.1 [9]
HDL-C mean (mg/dL)	52.8 ± 2.1 [4]	46.4 ± 4.9 [49]
ApoB mean (mg/dL)	120.5 ± 22.4 [5]	129.5 ± 25.2 [47]

PCSK9i indicates Proprotein convertase subtilisin/kexin type 9 inhibitor; SD, standard deviation; BMI, body mass index; LDL-C, low-density lipoprotein cholesterol; TC, total cholesterol; TG, triglyceride; HDL-C, high-density lipoprotein cholesterol; ApoB, apolipoprotein B.

^a^ Values are expressed as mean ± SD [n] unless otherwise noted. [n] indicates the number of trials reporting the characteristic. If no SD is reported, it is because only one study reported the characteristic. For trials that reported baseline characteristics separately for two arms (e.g., different statins), the average of the two values is presented in the table.

^b^ Race-ethnicity percentages do not add up to 100% as they represent averages across the studies, each with different sample sizes and distributions.

^c^ Up to 20 data points analyzed, comprised of 4 active arms from an individual participant-data level pooled analysis of 37 trials plus 16 active arms from 12 trials included in the pooled analysis.

Baseline lipid parameters were mostly similar between the high intensity statin and PCSK9i trial patients, including LDL-C,TC, HDL, and ApoB (Table 2). For TG, only one PCSK9i trial reported data and the value was lower than for the high intensity statin trials.

### 3.4 Meta-Analysis results

#### 3.4.1 Low-Density lipoprotein cholesterol (LDL-C)

The baseline mean LDL-C (mean ± SD) levels were similar between the PCSK9i trials and the high-intensity statin trials (160.3 ± 40.6 mg/dL vs. 147.6 ± 34.6 mg/dL). The PCSK9is demonstrated significantly greater reductions in LDL-C compared to all high-intensity statin regimens combined. The mean percent change in LDL-C (±SE) for PCSK9is was −52.4 ± 1.6%, whereas for all statin regimens combined, it was −46.6 ± 2.1% (*p* = 0.03).

**Figure 2:**
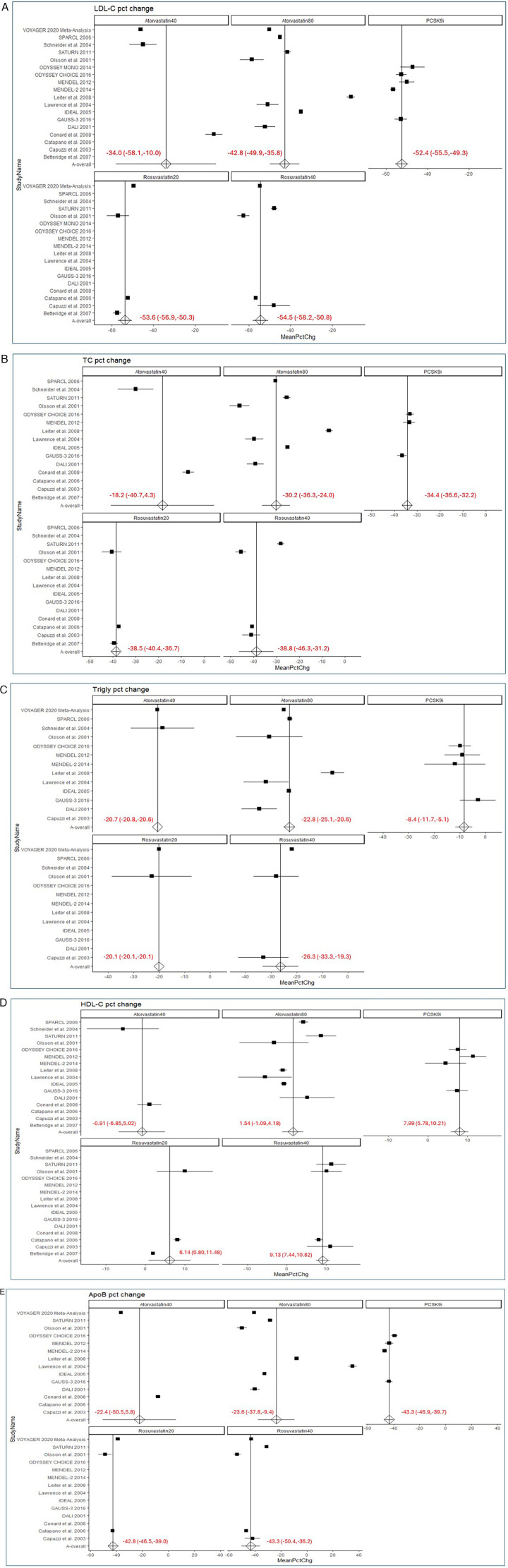
Forest plots. **(A)** Forest plots of mean percent change in LDL-C. **(B)** Forest plots of mean percent change in TC. **(C)** Forest plots of mean percent change in TG. **(D)** Forest plots of mean percent change in HDL-C. **(E)** Forest plots of mean percent change in Apo-B. Forest plots of relation of high-intensity statins and PCSK9 inhibitors to changes in serum lipid parameters. In each panel A-E, the upper left forest plot shows effect of atorvastatin 40 mg daily, the upper middle forest plots shows effect of atorvastatin 80 mg daily, the lower left forest plot shows effect of rosuvastatin 20 mg daily, the lower right forest plot shows effect of rosuvastatin 40 mg daily, and the upper right forest plot shows the effect of PCSK9 inhibitors. A, LDL cholesterol; B, Total cholesterol; C, Triglyceride; D, HDL cholesterol; E, ApoB. MeanPctChg = mean percent change. To retain the robustness of individual participant level-data compared with study-level data, the VOYAGER meta-analysis is shown as a single row and not decomposed into its 37 contributing trials.

Subgroup analysis of individual statin agents found that PCSK9is were superior to atorvastatin but comparable to rosuvastatin in reducing LDL-C (Figure 2A). The mean percent change in LDL-C (±SE) was −40.5 ± 3.0% for atorvastatin and −54.1 ± 1.3% for rosuvastatin (*p* < 0.001). Within the atorvastatin subgroups, LDL-C reductions were −34.0 ± 12.3% for atorvastatin 40 mg and −42.8 ± 3.6% for atorvastatin 80 mg (*p* < 0.001). In the rosuvastatin subgroups, LDL-C reductions were −53.6 ± 1.7% for rosuvastatin 20 mg and −54.5 ± 1.9% for rosuvastatin 40 mg (*p* = 0.394).

#### 3.4.2 Total cholesterol (TC)

The baseline TC levels (mean ± SD) were similar between the PCSK9i trials and the high-intensity statin trials (247.9 ± 40.8 mg/dL vs. 220.6 ± 30.3 mg/dL). PCSK9is demonstrated a similar mean percent reduction in TC (±SE) compared to high-intensity statins (–34.4 ± 1.1% vs. −32.6 ± 2.4%, *p* = 0.479).

When comparing individual statin agents, PCSK9is did not show a significantly different reduction in TC compared to atorvastatin 40 mg (−18.2 ± 11.5%, *p* = 0.160) or 80 mg (−30.2 ± 3.1%, *p* = 0.201), although reduction was greater compared with the two statin subgroups combined (−27.6 ± 3.0%, *p* = 0.03; Figure 2B). Conversely, PCSK9is were slightly less effective than rosuvastatin in reducing TC (–38.8 ± 1.7%, *p* = 0.04). This difference was statistically significant for rosuvastatin 40 mg (–38.5 ± 1.0%, *p* = 0.005) but not for rosuvastatin 20 mg. No significant difference in TC reduction was observed between PCSK9is and atorvastatin 40 mg or 80 mg.

#### 3.4.3 Triglyceride (TG)

The baseline TG level (mean ± SD) for the high intensity statin trials was found to be 154.1 ± 91.0 mg/dL. Among PCSK9i trials, only MENDEL (Monoclonal Antibody Against PCSK9 to Reduce Elevated LDL-C in Subjects Currently Not Receiving Drug Therapy For Easing Lipid Levels) 2012 reported baseline TG levels (59.9 mg/dL) (10). The mean percent reduction in TG (±SE) from these substantially different starting points suggested that PCSK9i monotherapy was less effective in reducing TG levels compared to high-intensity statins (−8.4 ± 1.7% vs. −22.3 ± 0.8%, *p* < 0.001; Figure 2C).

#### 3.4.4 High-Density lipoprotein cholesterol (HDL-C)

Baseline HDL-C levels (mean ± SD) were similar between the PCSK9i trials and the high-intensity statin trials (52.8 ± 2.1 mg/dL vs. 46.4 ± 4.9 mg/dL). PCSK9is resulted in a significantly greater mean percent increase in HDL-C (±SE) compared to high-intensity statins (8.0 ± 1.1% vs. 4.4 ± 1.0%, *p* = 0.02).

Subgroup analysis of individual statin agents showed that PCSK9is increased HDL-C significantly more than atorvastatin (1.1 ± 1.2%, *p* < 0.001) but were comparable to rosuvastatin (Figure 2D). Within the atorvastatin subgroup, PCSK9is led to a significantly greater percent change in HDL-C compared to both atorvastatin 40 mg (1.5 ± 1.3%, *p* = 0.006) and atorvastatin 80 mg (−0.9 ± 3.0%, *p* < 0.001). No significant difference was observed between PCSK9is and rosuvastatin 20 mg or 40 mg.

#### 3.4.5 Apolipoprotein B (apoB)

The baseline ApoB level (mean ± SD) for PCSK9is was 120.5 ± 22.4 mg/dL, lower than that of the high intensity statin trials at 129.5 ± 25.2 mg/dL. PCSK9is showed a significantly greater mean percent reduction (±SE) in ApoB levels compared to that of all high intensity statin regimens combined (−43.3 ± 1.8% vs. −32.8 ± 3.3%, *p* = 0.004).

Subgroup analysis of individual statin agents showed that PCSK9is resulted in a significantly greater reduction in ApoB compared to atorvastatin (–23.3 ± 5.9%, *p* = 0.001) but were comparable to rosuvastatin (Figure 2E). Within the atorvastatin subgroup, PCSK9is were more effective than atorvastatin 80 mg at reducing ApoB (−23.6 ± 7.3%, *p* = 0.008). No significant difference was observed between PCSK9is and atorvastatin 40 mg, rosuvastatin 20 mg, and rosuvastatin 40 mg.

## 4 Discussion

In this systematic meta-analysis, PCSK9i monotherapy, compared to high intensity statins as a class, demonstrated significantly greater reductions in LDL-C and ApoB, similar reduction in total cholesterol, and greater increase in HDL. Considering individual high intensity statin agents, PCSK9i monotherapy generally showed superiority over atorvastatin in modifying lipid profiles, including atorvastatin 80 mg, but comparable efficacy to rosuvastatin. Effects on TG could not be reliably characterized as there was only a single PCSK9i trial providing data and it had a disparate baseline TG level starting point.

The findings of this study are perforce consistent with the prior individual studies included in the meta-analysis. They also align generally with previous studies which have consistently shown that PCSK9is are capable of lowering LDL-C by approximately 60% and are a well-tolerated and effective therapy for reducing atherosclerotic cardiovascular disease when used in combination with statins (4). Large clinical trials, including FOURIER and ODYSSEY, have confirmed that PCSK9is are able to significantly lower LDL-C levels when used in combination with maximally tolerated statin treatment. Additionally, prior meta-analyses have shown that rosuvastatin is more effective than atorvastatin in reducing LDL-C, with rosuvastatin 40 mg showing the greatest reductions (22). This is consistent with our subgroup analysis, which found that PCSK9is were superior to atorvastatin but comparable to rosuvastatin in lowering LDL-C.

This study indirectly comparing agent potency by comparing PCSK9i vs. control and statin vs. control studies also align with the one study we have been able to identify that directly compared PCSK9is vs. statins in the same study population, albeit in healthy normolipidemic men rather than patients with cardiovascular disease. A factorial design evaluated the effects of high-dose atorvastatin alone, evolocumab, alone, and both on lipoprotein metabolism and found that both agent classes increased the catabolism of LDL particles while evolocumab, but not atorvastatin, also decreased the production of LDL particles (27). Evolocumab yielded a greater reduction in LDL than atorvastatin. A follow-up analysis of this trial found that triglyceride-rich lipoprotein metabolism was improved by high-dose atorvastatin, but not by high-dose evolocumab (28). The current study indicates that lipid profiles in hyperlipidemic patients with cardiovascular disease similarly differentially respond to PCSK9i and high intensity statin therapy as healthy normolipidemic individuals.

This study's findings have important immediate clinical implications for the treatment of hyperlipidemia, particularly for patients who are unable to tolerate statins. The high efficacy of PCSK9is in improving LDL-C, HDL-C, TC, comparable to rosuvastatin and superior to atorvastatin, indicates that PCSK9is are an effective lipid-level modifying treatment for statin-intolerant patients. These results support the current use of PCSK9is as valuable second-line agents for patients with total statin intolerance. The findings also have future clinical implications for the coming era when PCSK9is go off patent and become price-competitive with statins, currently estimated to occur between 2027–2031 (29, 30). At that time, it would be reasonable to consider both PCSK9is as first-line agents for lipid control. PCSK9is may be preferred over atorvastatin because of greater lipid-lowering effect. Treatment selection between PCSK9is and rosuvastatin may be guided by factors other than lipid-lowering intensity, such as patient preference between daily oral therapy versus injections every 2 to 4 weeks.

### 4.1 Limitations

There are limitations to this study that should be considered. First, while most patient baseline characteristics were similar in the PCSK9i trials vs. the statin trials, the lower prevalence of diabetes and tobacco usage in the PCSK9i trials may have influenced the observed treatment effects. Second, the sample size for high-intensity statin trials (*n* = 19,603) was substantially larger than that for PCSK9i trials (*n* = 766). Additionally, among the PCSK9i trials, only the MENDEL trial reported baseline TG levels, limiting the ability to conduct a robust statistical comparison between PCSK9is and high intensity statins on this lipid parameter (10). Given this single available trial, the mean percent reduction in TG and comparisons to PCSK9is should be interpreted cautiously. Third, this analysis focuses on serum lipid level changes rather than clinical endpoints such as myocardial infarction or stroke, reflecting that trials focused on quantifying lipid-lowering effects generally had short-term follow-up periods and therefore often did not report cardiovascular event frequencies. While reductions in LDL-C, TC and ApoB are well-established markers for cardiovascular risk reduction, it is also important to understand clinical benefits and potential adverse effects of PCSK9i monotherapy compared to statin monotherapy. Large-scale trials have demonstrated the clinical efficacy of both therapies in reducing major adverse cardiovascular events, but direct comparisons between the two agents remain lacking. Future studies should examine how changes in lipid parameters translate into clinical outcomes, providing a more comprehensive understanding of the long-term usage of PCSK9i monotherapy relative to statins.

### 4.2 Conclusion

In conclusion, this study-level meta-analysis aggregating 5 PCSK9i trials and 49 high intensity statin trials determined that PCSK9i monotherapy is superior to high-dose atorvastatin and comparable to high-dose rosuvastatin in improving LDL-C, HDL-C, TC, and ApoB, though inferior in reducing TG. These findings indicate that PCSK9is are currently highly useful second-line agents in patients with total statin intolerance. Further, with the upcoming expiration of patent protections, PCSK9is may also become important first-line treatment options for patients requiring advanced lipid-lowering therapy.

## Data Availability

The datasets analyzed in this study were derived from previously published trials, which are cited in the article. Extracted data used for analysis are available from the corresponding author upon request.
