# Supplementary material for: The lipid lowering efficacy of PCSK9 inhibitors alone vs. statins alone: a meta-analysis
Source: Front Cardiovasc Med. 2026 Feb 17;13:1769430. doi: 10.3389/fcvm.2026.1769430 (PMC12954586; doi:10.3389/fcvm.2026.1769430)
Supplement: Supplementary file 1 [file Table1.docx]

Supplementary Material

**Supplementary Table 1 (S1): Baseline Characteristics and Lipid Parameters in PCSK9i vs High-Intensity Statin Trials (Sample-Size Weighted Means)**

| **Characteristic** | **PCSK9i Trials**  **(5 trials; 766 pts) [n]*** | **High Intensity Statin Trials**  **(49 trials; 19,603 patients) [n]*** |
| --- | --- | --- |
| Age, mean | 56.8 [5] | 60.5 [48] |
| Female (%) | 56.5 [5] | 37.1 [48] |
| BMI (kg/m²) | 29.1 [4] | 27.9 [7] |
| **Race-Ethnicity**† | | |
| White (%) | 88.5 [5] | 74.0 [43] |
| Black (%) | 6.3 [3] | 5.5 [40] |
| Asian (%) | 7.8 [1] | 5.6 [38] |
| Other Race (%) | 1.8 [2] | 4.4 [38] |
| Hispanic (%) | 6.4 [1] | 4.3 [1] |
| **Vascular Risk Factors** | | |
| Hypertension (%) | 42.2 [3] | 44.0 [6] |
| Diabetes Mellitus (%) | 5.1 [4] | 27.5 [47] |
| Tobacco Use (%) | 11.9 [2] | 21.5 [5] |
| **Lipid Parameters** | | |
| LDL-C mean (mg/dL) | 160.4 [5] | 150.6 [49] |
| TC mean (mg/dL) | 256.6 [4] | 206.8 [12] |
| TG mean (mg/dL) | 59.9 [1] | 147.4 [9] |
| HDL-C mean (mg/dL) | 52.3 [4] | 48.0 [49] |
| ApoB mean (mg/dL) | 120.4 [5] | 142.6 [47] |

*Values are expressed as mean [n] where [n] indicates the number of trials reporting the characteristic. For trials that reported baseline characteristics separately for two arms (e.g., different statins), the average of the two values is presented in the table.

†Race-ethnicity percentages do not add up to 100% as they represent averages across the studies, each with different sample sizes and distributions.

PCSK9i indicates Proprotein convertase subtilisin/kexin type 9 inhibitor; BMI, body mass index; LDL-C, low-density lipoprotein cholesterol; TC, total cholesterol; TG, triglyceride; HDL-C, high-density lipoprotein cholesterol; ApoB, apolipoprotein B.
